# The imperative for regulatory oversight of large language models (or generative AI) in healthcare

**DOI:** 10.1038/s41746-023-00873-0

**Published:** 2023-07-06

**Authors:** Bertalan Meskó, Eric J. Topol

**Affiliations:** 1 The Medical Futurist Institute, Budapest, Hungary; 2 Department of Behavioural Sciences, Semmelweis University, Budapest, Hungary; 3 Scripps Research Translational Institute, Scripps Research, La Jolla, CA USA

**Keywords:** Health policy, Information technology, Policy

## Abstract

The rapid advancements in artificial intelligence (AI) have led to the development of sophisticated large language models (LLMs) such as GPT-4 and Bard. The potential implementation of LLMs in healthcare settings has already garnered considerable attention because of their diverse applications that include facilitating clinical documentation, obtaining insurance pre-authorization, summarizing research papers, or working as a chatbot to answer questions for patients about their specific data and concerns. While offering transformative potential, LLMs warrant a very cautious approach since these models are trained differently from AI-based medical technologies that are regulated already, especially within the critical context of caring for patients. The newest version, GPT-4, that was released in March, 2023, brings the potentials of this technology to support multiple medical tasks; and risks from mishandling results it provides to varying reliability to a new level. Besides being an advanced LLM, it will be able to read texts on images and analyze the context of those images. The regulation of GPT-4 and generative AI in medicine and healthcare without damaging their exciting and transformative potential is a timely and critical challenge to ensure safety, maintain ethical standards, and protect patient privacy. We argue that regulatory oversight should assure medical professionals and patients can use LLMs without causing harm or compromising their data or privacy. This paper summarizes our practical recommendations for what we can expect from regulators to bring this vision to reality.

## Introduction

The rapid advancements in artificial intelligence (AI) have led to the development of sophisticated large language models (LLM) such as OpenAI’s GPT-4 and Google’s Bard^1,2^. The unprecedented popularity of ChatGPT, GPT-4’s predecessor released in November 2022, is reflected by the most rapid uptake of users - 100 million in 2 months - for any new technology.

This rapid growth sparked global debates about the role such conversational chatbots could play in healthcare and the practice of medicine. Diverse applications of LLMs have appeared including facilitating clinical documentation; creating discharge summaries; generating clinic, operation, and procedure notes; obtaining insurance pre-authorization; summarizing research papers; or working as a chatbot to answer questions for the patients with their specific data and concerns. LLMs can also assist physicians in diagnosing conditions based on medical records, images, laboratory results, and suggest treatment options or plans. At the same time, patients can potentially become more autonomous than with prior search methods by obtaining individualized assessment of their data, symptoms, and concerns.

Systematic reviews highlighted other potential benefits too such as improved scientific writing, enhancing research equity, streamlining the healthcare workflow, cost saving, and improved personalized learning in medical education^3,4^.

Given the potential implications on patient outcomes and public health, it is imperative to consider how these new AI-based tools should be regulated. The regulation of these LLMs in medicine and healthcare without damaging their promising progress is a timely and critical challenge to ensure safety, maintain ethical standards, pre-empt unfairness and bias, and protect patient privacy. Whatever concerns have been previously recognized with AI are now markedly amplified with the multipotency of LLMs.

This paper explores the potential risks and benefits of applying LLMs in healthcare settings and argues for the necessity of regulating LLMs differently than AI-based medical technologies that are already on the market to mitigate potential harm and maintain public trust in these breakthrough technologies.

## LLMs differ from already regulated AI-based technologies

LLMs differ significantly from prior deep learning methods in terms of their scale, capabilities, and potential impact. Here we outline the key characteristics of LLMs that set them apart from traditional deep learning techniques.

### Scale and complexity

LLMs are trained on massive datasets and utilize billions of parameters, resulting in unprecedented complexity. This level of sophistication requires regulatory oversight that takes into account the challenges associated with interpretability, fairness, and unintended consequences. Moreover, LLMs use tokens that can be words, subwords, or even characters as the smallest units of text used to represent and process language during the training and generation processes. Tokenization is a crucial step in natural language processing (NLP) and allows LLMs to efficiently analyze and generate text, as these models are designed to process sequences of tokens rather than entire sentences or paragraphs. Currently, tokenization is not covered by healthcare regulators.

### Hardware requirements

LLMs require massive computational resources in terms of floating-point operations per second (FLOPs) and graphics processing unit (GPU) usage compared to previous deep learning models due to their large scale, extensive training data, a type of neural network model designed for NLP tasks called the Transformer architecture, and the need for fine-tuning.

### Broad applicability

Unlike specialized deep learning models that were trained to address a specific medical issue or clinical need, LLMs possess versatile capabilities that span various domains, such as healthcare, finance, and education. As a result, a one-size-fits-all regulatory framework is ill-suited for LLMs, and oversight must be adaptable to address diverse industry-specific concerns.

### Real-time adaptation

LLMs can adapt their responses in real-time, based on user input and evolving contexts. This dynamic behavior demands that regulatory oversight incorporates continuous monitoring and evaluation mechanisms to ensure responsible usage and adherence to ethical guidelines. This is similar to what adaptive AI-based medical technologies would require from regulators.

### Societal impact

The widespread adoption of LLMs has the potential to fundamentally transform various aspects of society. Consequently, regulatory oversight must address not only the technical aspects of LLMs but also their broader ethical, social, and economic implications.

### Data privacy and security

LLMs’ reliance on extensive training data raises concerns related to data privacy and security. Regulatory oversight should establish robust frameworks to protect sensitive information and prevent unauthorized access or misuse of these powerful models.

These unique characteristics of LLMs necessitate a tailored approach to regulatory oversight. Such an approach must be adaptive, holistic, and cognizant of the diverse challenges and potential consequences that LLMs present, ensuring their responsible and ethical use across various domains.

## The FDA’s Pre-LLM oversight of AI

The United States’ Food And Drug Administration (FDA) has been leading the global discussions on regulatory oversight and has been a prominent example in providing regulations about emerging technologies from 3D printed medications to AI-based medical tools^5^.

With the increasing adoption of digital health technologies, the FDA started regulating Software as a Medical Device (SaMD) that refers to software solutions that perform medical functions and are used in the prevention, diagnosis, treatment, or monitoring of various diseases or conditions.

As a continuation of that approach, the FDA has been adapting its regulatory framework to specifically address AI and machine learning (ML) technologies in medical devices^6^. The FDA released a discussion paper that outlined their potential regulatory approach tailored to AI and ML technologies used in medical devices^7^. The discussion paper proposed a total product lifecycle (TPLC) approach to regulating AI/ML-based SaMD, which focuses on the continuous monitoring and improvement of these technologies throughout their lifespan. The proposed framework also emphasized the importance of transparency, real-world performance monitoring, and clear expectations for modifications and updates to AI/ML algorithms.

Currently, the FDA does not have specific categories exclusively for AI-based technologies but evaluates them within the existing regulatory framework for medical devices^8^. They classify such devices into three main categories based on their level of risk:

- Class I (Low risk): These devices pose the least risk and are subject to general controls, such as registration and listing, labeling, and good manufacturing practices. Examples of Class I devices include non-powered surgical instruments and dental floss. Some low-risk AI-based medical technologies may fall under this category, depending on their intended use.
- Class II (Moderate risk): These devices carry a higher level of risk than Class I devices and are subject to both general controls and special controls, such as performance standards, postmarket surveillance, or specific labeling requirements. Examples of Class II devices include infusion pumps, surgical drapes, and powered wheelchairs. Many AI-based medical technologies, such as diagnostic imaging systems, may fall under this category.
- Class III (High risk): These devices pose the highest risk and are subject to general controls, special controls, and premarket approval (PMA). Class III devices often support or sustain human life, are of substantial importance in preventing impairment of human health, or present a potential unreasonable risk of illness or injury. Examples of Class III devices include implantable pacemakers, artificial heart valves, and some AI-based technologies used in critical medical decision-making.

AI-based medical technologies may also be subject to the FDA’s Digital Health Software Precertification (Pre-Cert) Program, which is designed to streamline the regulatory process for SaMD, including AI-based technologies.

A milestone in that process was the release of their database of specifically AI-based medical technologies with regulatory approvals in 2021^9^. As of April, 2023, 521 devices are included in that database. The most popular categories are radiology, cardiovascular and hematology with 392, 57 and 15 devices, respectively. The vast majority (96%) were approved with a 510(k) clearance, while 18 (3.5%) received de novo pathway clearance and 3 (0.5%) premarket approval (PMA) clearance.

As other papers have pointed out, only a few of these devices were tested in randomized controlled trials (RCTs) trials; and only a limited number of studies have used external validation, prospective evaluation and diverse metrics to explore the full impact of AI in real clinical settings, and the range of assessed use cases has been relatively narrow with no or very little transparency^10^.

In summary, while there has been progress in regulating AI, the FDA has not been able to solve the regulation of two advanced technological issues that are related but not the same. One is about regulating adaptive algorithms that can adjust its parameters or behavior based on the input data or its performance on a specific task. This adaptability allows the algorithm to improve its performance over time or respond to changing conditions.

The other one is related to the so-called autodidactic function in deep learning. It refers to the ability of a system to teach itself without direct supervision, an approach that often requires unsupervised or self-supervised learning, where the model learns patterns and representations from the input data without relying on labeled examples. Such an autodidactic deep learning model can discover underlying structures and relationships in the data by optimizing its internal representations without explicit guidance.

## The LLM era in the practice of medicine

**Fig. 1:**
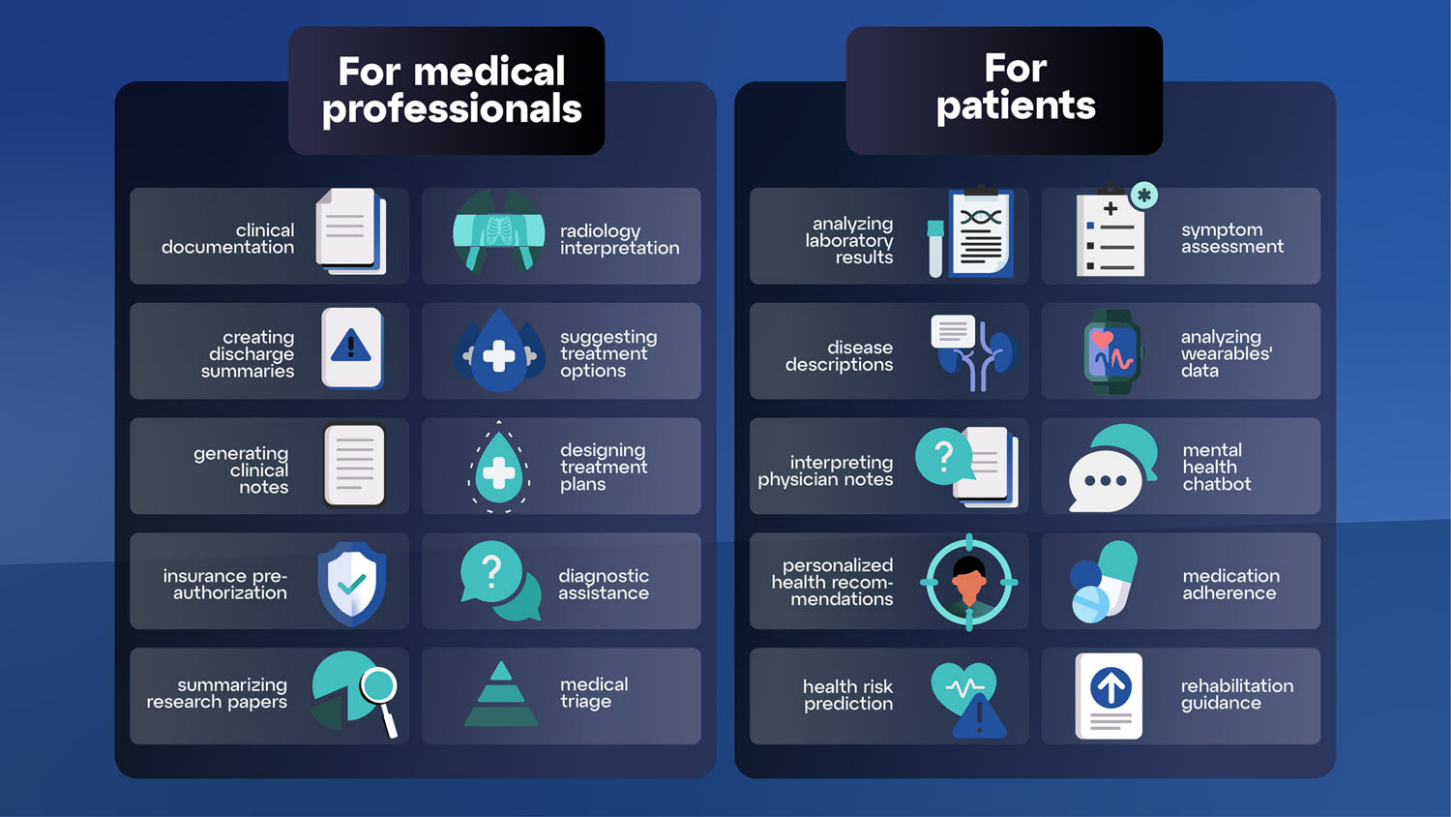
Ten examples of use cases of LLMs for medical professionals; and ten examples for patients.

To date, no LLM has had pre-training with the corpus of medical information or with millions of patient records, images, lab data, and office visit or bedside conversations. Details about the training of GPT-4, the most advanced LLM that was pubished in March 2023, have not been released. Nevertheless, LLMs have transformative potential, with use cases ranging from clinical documentation to providing personalized health plans^11^. Figure 1 describes 10 use cases for medical professionals and 10 for patients.

At the same time, the introduction of these models into healthcare leads to the amplification of risks and challenges.

It started posing a new challenge to physicians as patients arrive to the meeting with not only responses received after googling their symptoms but also from ChatGPT-like chatbots. There have been discussions about to what extent ChatGPT can be used for medical research and summarizing peer-reviewed papers when it only provides sources it based its responses on after specifically asking for it. Moreover, some of those sources have been reported to be made up^3^.

LLMs can sometimes "hallucinate" results, which refers to generating outputs that are not grounded in the input data or factual information. Such misinformation may be related to a diagnosis, treatment, or a recommended test. For the uninitiated, such outputs are conveyed with a high level of confidence and could easily be accepted by the prompter as truth—which has the potential to be dangerous. Whether it is due to incomplete or biased training data, its probabilistic nature or the lack of context; it poses a significant risk of providing unreliable or outright false answers in the medical setting that might have serious consequences.

Another issue, bias in medicine while using LLMs can affect clinical decision-making, patient outcomes, and healthcare equity. If the training data contains biases, such as underrepresentation of certain demographic groups, overemphasis on specific treatments, or outdated medical practices, LLMs may inadvertently learn and propagate these biases in its outputs. Biased outputs from GPT-4 may lead to incorrect diagnoses or suboptimal treatment recommendations, potentially causing harm to patients or delaying appropriate care.

GPT-4 brings the potentials and the risks to a new level. It will be able to read texts on images (including physicians’ hand-written notes), and analyze the content and context of images. Table 1 summarizes the key differences between the previous and the new version regarding healthcare-related and medical prompts. It shows that GPT-3 could handle simple prompts with general queries, while GPT-4 is able to analyze complex, multi-level prompts, and provide more sophisticated results such as case descriptions or research paper summaries.

**Table 1:** Differences between the depth and details of prompts for ChatGPT and GPT-4.

Prompts	ChatGPT	GPT-4
Prompt 1 – Diagnosing a patient with ambiguous symptoms	A patient presents with fatigue, weight loss, and occasional dizziness. What are some possible causes for these symptoms?	A 45-year-old male patient presents with a 3-month history of progressive fatigue, unintentional weight loss of 15 pounds, and episodes of dizziness. Please provide a differential diagnosis and suggest relevant diagnostic tests.
Prompt 2 – Treatment recommendations	What are some common treatments for type 2 diabetes?	A 55-year-old female with a recent diagnosis of type 2 diabetes has an HbA1c level of 8.5%. Outline a comprehensive treatment plan, including lifestyle modifications, pharmacological options, and follow-up monitoring.
Prompt 3 – Patient education	Explain high blood pressure in simple terms.	Create a patient-friendly educational handout on hypertension, including an overview of the condition, risk factors, symptoms, potential complications, and management strategies.
Prompt 4 – Reviewing medical research	Tell me about the benefits of exercise for mental health.	Summarize recent research findings on the relationship between physical activity and mental health outcomes, including potential mechanisms, types of exercise, and recommendations for various populations.
Prompt 5 – Clinical case scenario	Describe a patient with pneumonia.	Create a detailed clinical case scenario involving a 65-year-old patient presenting with community-acquired pneumonia, including history of present illness, relevant past medical history, physical examination findings, diagnostic test results, and treatment plan.

The application of GPT-4 in healthcare raises ethical concerns that warrant a regulatory framework. Issues such as transparency, accountability, and fairness need to be addressed to prevent potential ethical lapses. For instance, healthcare professionals and patients should be made aware of the AI’s involvement in the decision-making process and be provided with explanations for the AI’s recommendations.

Moreover, regulatory oversight can help ensure that AI-driven models do not perpetuate or exacerbate existing healthcare disparities. By mandating diverse and representative data sources, regulators can counteract potential biases within the AI’s training data, thus promoting fairness in the delivery of healthcare services.

The use of GPT-4 and ChatGPT in such environments calls for robust regulations to ensure the confidentiality and security of patient information. This could include specific guidelines for data anonymization, encryption, and secure storage, as well as measures to prevent unauthorized access or misuse of data by third parties.

As a sign of wide implementation, medical companies, digital health services and healthcare organizations have already started to implement ChatGPT into their core business. Examples include the Microsoft-owned Nuance as they decided to add GPT-4 AI to its medical note-taking tool; and a French startup called Nabla that claimed to be the first to build a tool using GPT-3 to help physicians do their paperwork^12,13^.

All these examples and challenges prompt regulatory bodies to not only start regulating LLMs as those models are being deployed, but to regulate them differently that AI-technologies currently on the market.

## The regulatory challenges of LLMs

Most LLMs have been released globally and no country-specific iterations are available requiring a global approach from regulators. It is also not clear what technical category LLMs will fall into from the regulatory perspective. However, based on the differences between LLMs and prior deep learning methods, a new regulatory category might be needed to address LLM-specific challenges and risks.

A regulatory body only has to design regulations for LLMs if either the developers of LLMs make claims that their LLM can be used for a medical purpose; or if LLMs are developed for, adapted, modified or directed toward specifically medical purposes. Even if currently widespread LLMs won’t fall into either category, the medical alternatives of LLMs specifically trained on medical data and databases probably will.

One prominent example is Med-PaLM that DeepMind and Google researchers have published about. In that study, authors proposed a framework for human evaluation of model answers along multiple axes including factuality, precision, possible harm, and bias. In addition, using a combination of prompting strategies, their model achieved 67.6% accuracy on the US Medical License Exam questions, surpassing prior state-of-the-art by over 17%. As human evaluation reveals key gaps in the responses provided by the LLM, they introduced instruction prompt tuning and the resulting model, Med-PaLM, performs encouragingly, but remains inferior to clinicians. Since then, GPT-4 could achieve an accuracy over 85% on the same exam^14^.

With the release of GPT-4 that can analyze not only texts but images, it can be expected that the model will grow to analyze uploaded documents, research papers, hand-written notes, sound, and video in the near future. (Table 2).

**Table 2:** A list of types of content forms that LLMs could analyze now and possible new versions in the future.

Type of content	Potential applications	Availability
text/conversations	chatbots, text analysis, documentation	Yes
image analysis	detecting the content and the context of images	In 2023
document/PDF analysis	analyzing research papers and creating summaries of documents	N/A
sound	voice-to-text applications and sound-based interactions	N/A
video	analyzing the content of videos and creating deepfakes	N/A

This underscores the notion that it is not enough to regulate current LLM models as the new iterations with those advanced capabilities can be expected to get implemented at a similar rate of the previous iterations. Without taking these future additions into consideration, a regulation that focuses on language models only could miss important updates by the time those updates become widely accessible.

Companies with approved devices that decide to implement LLMs into their services face an additional challenge. Namely, how will the FDA regulate an AI-based medical technology recently infused with LLM if the technology was already approved for medical uses? Table 3 summarizes the regulatory challenges.

**Table 3:** A list of regulatory challenges related to the rise of LLMs.

Regulatory challenge	Short description
Patient Data Privacy	Ensuring that patient data used for training large language models are fully anonymized and protected from potential breaches. This poses a significant regulatory challenge, as any violation could lead to serious consequences under privacy laws like HIPAA in the US.
Intellectual Property	If an LLM generates content similar to proprietary medical research or literature, it could lead to issues regarding intellectual property rights.
Medical Malpractice Liability	Determining who is responsible when an AI’s recommendations lead to patient harm. Is it the AI developers, the healthcare professionals who used it, or the institutions that adopted it?
Quality Control & Standardization	Regulation is required to ensure the reliability and consistency of AI-generated medical advice, which can vary based on the data used to train the AI.
Informed Consent	Patients need to be informed and give consent when AI tools are used in their healthcare management. This is challenging because it can be difficult for patients to fully understand the implications of AI use.
Interpretability & Transparency	Regulations need to ensure transparency about how decisions are made by the AI. This is particularly challenging with AI models that are often termed as "black boxes" due to their complex algorithms.
Fairness and Bias	Regulation is needed to prevent biases in AI models, which could be introduced during the training process using patient data. This can lead to disparities in healthcare outcomes.
Data Ownership	It can be challenging to define and regulate who owns the data that large language models learn from, especially when it comes to patient data.
Over-reliance on AI Models	Over-reliance on AI could lead to decreased human expertise and potential errors if the AI malfunctions or provides incorrect information. Regulations are needed to balance the use of AI and human expertise.
Continuous Monitoring & Validation	Ensuring the continuous performance, accuracy, and validity of AI tools over time and across different populations is a critical regulatory challenge.

There have been proposals about regulating LLMs, although those come from outside healthcare. In a working paper, Hacker et al. suggests a novel terminology to capture the AI value chain by differentiating between developers, deployers, professional and non-professional users, as well as recipients of LLM output. Authors also suggested four strategies to ensure that these models are trustworthy and deployed for the benefit of society at large. In details, regulation should focus on concrete high-risk applications, and not the pre-trained model itself, and should include (i) obligations regarding transparency, (ii) risk management, (iii) non-discrimination provisions, and (iv) content moderation rules^15^.

Mökander at al pointed out that existing auditing procedures fail to address the governance challenges posed by LLMs, and offered three contributions to fill that gap namely 1) establishing the need to develop new auditing procedures that capture the risks posed by LLMs; 2) outlining a blueprint to audit LLMs in feasible and effective ways by drawing on best practices from IT governance and system engineering; and 3) discussing the limitations of the prospect of auditing LLMs at all^16^.

Such potential solutions could serve as a benchmark for new regulations in healthcare. In either case, regulators and lawmakers need to act fast to keep track with the dynamics of the unprecedented evolution and progress of LLMs.

As a sign of the rising pressure on regulators, in March 2023, a group of prominent computer scientists and technology industry executives such as Elon Musk and Steve Wozniak called for “all AI labs to immediately pause for at least 6 months the training of AI systems more powerful than GPT-4”^17^. Their letter mentioned that “recent months have seen AI labs locked in an out-of-control race to develop and deploy ever more powerful digital minds that no one – not even their creators – can understand, predict, or reliably control. This pause should be public and verifiable, and include all key actors. If such a pause cannot be enacted quickly, governments should step in and institute a moratorium.”

Notable AI experts such as Andrew Ng objected the idea and instead, called for seeking a balance between the huge value AI is creating vs realistic risks. We agree that a moratorium cannot be implemented in practice unless governments step in; and “having governments pause emerging technologies they don’t understand is anti-competitive, sets a terrible precedent, and is awful innovation policy”^18^.

To reinforce our concerns, it is worthy of mention that Italy became the first Western country to temporarily block ChatGPT in April 2023 due to privacy concerns and the lack of proper regulation^19^.

## Conclusions

LLMs offer tremendous promise for the future of healthcare, but their use also entails risks and ethical challenges. By taking a proactive approach to regulation, it is possible to harness the potential of AI-driven technologies like LLMs while minimizing potential harm and preserving the trust of patients and healthcare providers alike.

Furthermore, LLMs could also become the first category of AI-based medical technologies that are regulated by implementing patient design, meaning, regulators would finally involve patients on the highest level of decision-making ensuring that these AI tools that are progressing at an incredibly fast pace will be regulated to address real-life clinical and patient needs^20^.

Here we summarize what we can expect regulators to do about bringing LLMs to the practice of medicine.

- Create a new regulatory category for LLMs as those are distinctively different from AI-based medical technologies that have gone through regulation already.
- Provide a regulatory guidance for companies and healthcare organizations about how they can deploy LLMs into their existing products and services.
- Create a regulatory framework that not only covers text-based interactions but possible future iterations such as analyzing sound or video.
- Provide a framework for making a distinction between LLMs specifically trained on medical data and LLMs trained for non-medical purposes.
- Similar to the FDA’s Digital Health Pre-Cert Program, regulate companies developing LLMs instead of regulating every single LLM iteration.

### Reporting summary

Further information on research design is available in the Nature Research Reporting Summary linked to this article.

## Supplementary information

Reporting Summary
